# Municipal water quantities and health in Nunavut households: an exploratory case study in Coral Harbour, Nunavut, Canada

**DOI:** 10.3402/ijch.v73.23843

**Published:** 2014-03-28

**Authors:** Kiley Daley, Heather Castleden, Rob Jamieson, Chris Furgal, Lorna Ell

**Affiliations:** 1 Centre for Water Resources Studies, Dalhousie University, Halifax, Canada; 2 School for Resources and Environmental Studies, Dalhousie University, Halifax, Canada; 3 Department of Process Engineering and Applied Science, Dalhousie University, Halifax, Canada; 4 Indigenous Environmental Studies Program, Trent University, Peterborough, Canada; 5 Coral Harbour, Nunavut, Canada

**Keywords:** Inuit, domestic water, water security, public health, overcrowded housing, built environment, communicable diseases, Canadian Arctic

## Abstract

**Background:**

Access to adequate quantities of water has a protective effect on human health and well-being. Despite this, public health research and interventions are frequently focused solely on water quality, and international standards for domestic water supply minimums are often overlooked or unspecified. This trend is evident in Inuit and other Arctic communities even though numerous transmissible diseases and bacterium infections associated with inadequate domestic water quantities are prevalent.

**Objectives:**

Our objective was to explore the pathways by which the trucked water distribution systems being used in remote northern communities are impacting health at the household level, with consideration given to the underlying social and environmental determinants shaping health in the region.

**Methods:**

Using a qualitative case study design, we conducted 37 interviews (28 residents, 9 key informants) and a review of government water documents to investigate water usage practices and perspectives. These data were thematically analysed to understand potential health risks in Arctic communities and households.

**Results:**

Each resident receives an average of 110 litres of municipal water per day. Fifteen of 28 households reported experiencing water shortages at least once per month. Of those 15, most were larger households (5 people or more) with standard sized water storage tanks. Water shortages and service interruptions limit the ability of some households to adhere to public health advice. The households most resilient, or able to cope with domestic water supply shortages, were those capable of retrieving their own drinking water directly from lake and river sources. Residents with extended family and neighbours, whom they can rely on during shortages, were also less vulnerable to municipal water delays.

**Conclusions:**

The relatively low in-home water quantities observed in Coral Harbour, Nunavut, appear adequate for some families. Those living in overcrowded households, however, are accessing water in quantities more typically seen in water insecure developing countries. We recommend several practical interventions and revisions to municipal water supply systems.

Access to adequate quantities of in-home water has a protective effect on human health and well-being, as well as the potential to improve poor health situations (1). Despite this, public health research and interventions are frequently narrowed towards the quality of water, and international standards for domestic water supply minimum are often overlooked or unspecified (1). This trend is evident in Inuit and other northern communities, as most research has primarily focused on water quality or the effects of other issues on water such as climate change even though numerous communicable diseases and bacterium infections with partial relation to inadequate sanitation and hygiene – water quantity issues – are prevalent (2–5). For instance, rates of tuberculosis are known to be much higher in Inuit communities than the Canadian average (6). *Helicobacter pylori* (*H. pylori*) and methicillin-resistant *Staphylococcus aureus* (MRSA) infections are also of concern (7–9). *H. pylori* is a bacterium present in the human gastrointestinal tract. Although most people are asymptomatic, infection is more prevalent in developing countries and some Indigenous populations (7,8). *H. Pylori* infection has been linked with chronic enteric illness, stomach ulcers and cancer (7). MRSA is a bacterium causing skin and other infections that are difficult to treat with standard types of antibiotics (9). Increased risk of human-to-human transmission of all three of these infections has been associated with settlements dealing with overcrowded housing and in-home water supply shortages (6,10,11).

More recently, research frameworks that expand the concept of water security beyond quality to also include components of availability, access and stability over time have begun to emerge (12–14). Subsequently some northern health research has begun to acknowledge the importance of ample in-home water quantities but, with the exception of a multiple site case study of rural Northwest Alaskan villages, it has not been a central focus (15). One recent study from Russia, for example, does provide the water consumption rates of the country's Arctic communities, yet the discussion of the results is weighted predominantly towards water quality (16). Another study from Canada alludes to the potential health risks associated with domestic water supply shortages in northern communities however the authors’ main objective is the detection of potentially water-borne diarrhoeal pathogens, again a water quality issue (17). Given the minimal published research available to understand the connections between in-home water supply and health in Arctic communities, we conducted an exploratory case study of the current water–health relationship in a remote Inuit hamlet in Nunavut. The objective was to conceptualize the pathways by which municipal water systems and services may be impacting health at the household and family levels, with consideration given to the underlying social and environmental determinants shaping health in the region.

## Background

### Water systems and services in Nunavut

During the pre-permanent settlement period, prior to the 1950s, Canadian Inuit retrieved drinking water, or ice for melting, directly from the source. Wastewater was typically dispensed at safe distances from preferred drinking water sources and shelters. During the early settlement-era, beginning in the 1950s, variations of individual-haul systems, locally referred to as “barrel and honey bucket systems,” became common in many permanent settlements in the region and remained in use into the 1980s (18). Residents individually retrieved their own water from nearby lakes or rivers and typically stored it within a large barrel. Household wastewater was usually collected in a large pail – “the honey bucket” – and removed from the home (18).

**Fig. 1:**
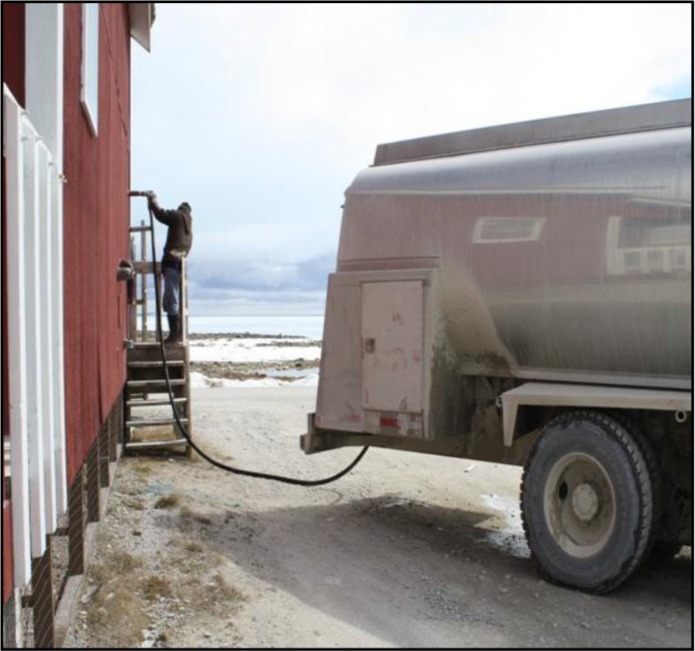
Tanker truck delivering potable water to house (photo credit: first author).

**Fig. 2:**
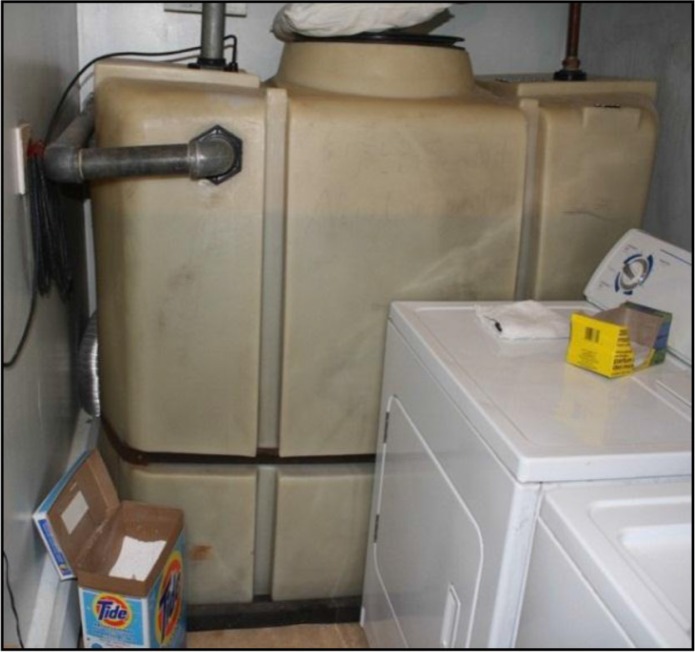
Potable water storage tank inside house, approximate capacity of 1,200 litres (photo credit: first author).

Currently, the source water used in Nunavut's municipal water systems is usually drawn from lakes or rivers and piped to a central holding reservoir where it undergoes chlorination for microbial control before delivery to residents for domestic use. Aside from chlorination, water does not typically receive any additional chemical or physical filtrations, which are methods of removing harmful naturally occurring contaminants from source waters, and are commonly used in much of southern Canada. Extensive underground piped water systems, which are used to distribute water from the central reservoir to homes and buildings in most urban areas of southern Canada, are not suitable for the cold climate and economic conditions in Nunavut (19). Permafrost also negates the possibility of individual water wells and buried septic systems, which are common options in many of southern Canada's rural settings (19). Instead, a trucked water system is employed in much of northern Canada. Tanker trucks deliver potable (suitable for drinking) water to storage tanks inside dwellings (Figs. 1 and 2).

Household wastewater is stored in a separate tank and pumped out using specialized trucks. The domestic wastewater is then removed to designated stabilization ponds and wetlands (wastewater treatment areas), which are typically located near solid waste disposal areas outside the community to prevent hydrologic connectivity with municipal drinking water sources. In most communities, if systems are functioning optimally, both water delivery and wastewater pump-out services are provided daily or at least 3 days per week to each home.

During the transition period from individual-haul to trucked water systems in Canada's northern settlements, 30 litres of water per capita per day was found to be the minimum amount required for public health protection in the region, while 65 litres per capita per day was determined as the amount of water necessary to reduce excessive incidence of gastrointestinal and skin disease (20). Based on this information, an augmented 100 litres per capita per day was recommended as a baseline design value for trucked water supply systems in Arctic communities during the 1990s and is still used today (21).

### Case context: Coral Harbour, Nunavut, Canada

**Fig. 3:**
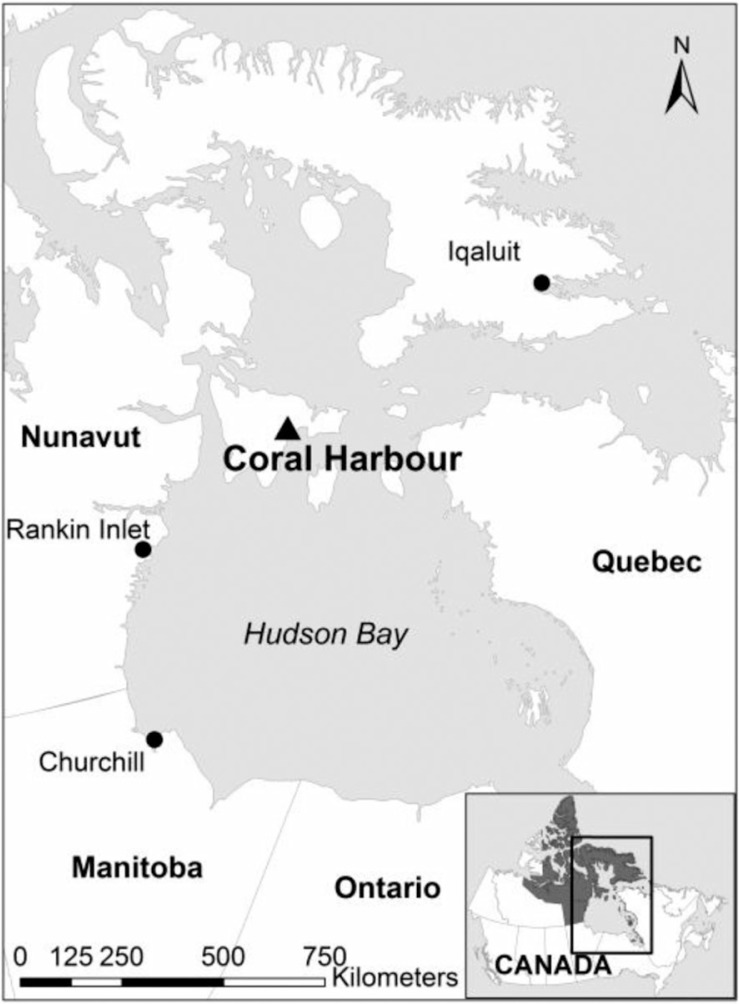
Locator map of case study site Coral Harbour, Nunavut, Canada.

Coral Harbour (latitude 64.137°N, longitude 83.167°W) is a remote fly-in community located in Nunavut's Kivalliq Region on Southhampton Island, north of Hudson Bay (Fig. 3). Inuit have inhabited this region for millennia, practicing a hunting and gathering-based subsistence lifestyle. In the 1950s and 1960s, the Government of Canada increased their presence on the Island, building a small school and clinic. During this time, nomadic Inuit began to settle permanently in the central community of Coral Harbour and access the education and health care services being offered (22).

The current population of Coral Harbour is approximately 850, with 95% of the residents identifying themselves as Inuit (23). Unlike many other non-Indigenous rural and remote areas of Canada, Nunavut's population is growing (24). Coral Harbour's population grew 8.5% since 2006, comparable to Nunavut's overall population increase of 8.3% and larger than Canada's overall growth of 5.9% during the same period (23). Demographically, Coral Harbour is a young community, with a median age of 21.8 years. Again, this is comparable to Nunavut's median age of 24.1 but in noteworthy contrast to Canada's median age of 40.6 years of age (23). In Coral Harbour, large families are common as are multiple generations or extended families residing together. Of the 205 private dwellings in the community, the reported average number of persons per household is 4.0, significantly larger than the Canadian average of 2.5 (23). In reality, the number of people residing in many homes in Coral Harbour may be larger still. The Territory of Nunavut faces serious overcrowding issues. “Hidden homelessness” is a term increasingly used to describe the scenario unfolding in Nunavut wherein those who do not have a home, live temporarily in others’ dwelling (25,26). Because economic opportunities in the community are limited there is no tax base, therefore, the municipality relies on funding assistance from territorial and federal levels of government. Publicly-funded housing comprises approximately two-thirds of the homes in Coral Harbour and they vary in their state of repair, similar to the situation throughout Nunavut (26). Dwellings in Coral Harbour consist of single houses, semi-detached houses and row houses. Generally, single houses are fitted with a 1,200 litre potable water holding tank, although dependent on the age of the building the tank size may vary. Semi-detached houses and row houses have similar size water tanks. However, unlike single houses, these buildings often include shared-access tanks, which are located in an insulated area underneath the structure, making it difficult to visually monitor the level of remaining water.

A small health centre in the community is staffed by three or four permanent and rotational nurses, who are typically from southern Canada, along with local community health representatives and administrative staff including interpreters. There are no permanent physicians. Family doctors, some specialists (e.g. dentists and optometrists) and public health officers make scheduled visits to the community a few times per year. Consultation with, and patient transfer via air to, full service hospitals in major centres such as Iqaluit (over 500 km) or Winnipeg (over 1,000 km) is the norm despite the considerable cost and distance. Despite these challenges, Coral Harbour is a proud, supportive Inuit community and strong ties to tradition remain. Nearly 80% of the population speak Inuktitut at home (27), with English as a second language. Travelling on the land and sea to seasonal campsites or cabins for fishing and hunting remains integral to the lifestyle of residents.

## Methods

The findings described in this paper are based on one component of a large municipal water and wastewater services research project underway at Dalhousie University in partnership with the Government of Nunavut. Through its involvement in earlier stages of the large project, Coral Harbour was identified as a potential site to conduct an exploratory case study of the effects of in-home water supply on health. We visited Coral Harbour to further gauge interest in the topic, need for the research, and desired level of community involvement. This preliminary visit included discussion and meetings with senior municipal staff, elected officials, health centre staff, water system operators and residents. Input gathered during this process was presented to the Municipal Hamlet Council and permission was subsequently granted to conduct the study in the community. The Hamlet also provided administrative support and information about municipal water and wastewater operations when requested. A Community Research Liaison (fifth author) was hired to assist with the participant recruitment, data collection and analysis stages of the project.

### Participant recruitment, data collection and data analysis

A total of 37 interviews were conducted. Nine interviews were with key informants and 28 interviews were with residents. Key informants were purposefully recruited based on their ability to provide insight on water and health topics within Coral Harbour and the Territory of Nunavut. These participants included water system operators, primary health care providers, public health officers and environmental organizations’ staff members. Some key informants were based in Coral Harbour and others were located across the territory.

The 28 resident participants were recruited using a combination of purposive and opportunistic sampling. The purposively recruited participants were selected based on their expressed interest in water and health issues within their community. The range of age, gender and family size characteristics among the participants provided diversity within the sample (28). Opportunistic sampling allowed for flexibility and inclusion of additional participants on the basis of interesting leads uncovered during early stages of the research (29). The Community Research Liaison led the resident participant recruitment process using posters, radio messages and word-of-mouth contact.

Key informant interview questions were related to their occupation or role, and the understanding that position provided them regarding water and health related issues in the community. Interviews with residents pertained to water usage patterns, opinions of water treatment and delivery in the community, and water-related health issues. Interviews ranged in length from 20 to 90 minutes. The semi-structured interview guide used consisted of 25 questions and included several open-ended questions giving the participant opportunity to discuss water and health issues that they deemed important but were not anticipated during the research design (30). During interviews, participants spoke in the language of their choice, either Inuktitut or English. Of the 37 interviews, 33 were conducted in English and four in Inuktitut. The Community Research Liaison provided translation during the Inuktitut interviews. As she was translating, she asked clarifying questions of both the English-speaking interviewer (first author) and Inuktitut-speaking participants as needed in an effort to provide as accurate a translation as possible. Interview data were supplemented with secondary data from public documents relating to water usage figures, land planning and municipal water infrastructure.

Data were analysed using qualitative content analysis and constant comparative methods (31,32). The first author created a code book consisting of a combination of theory-driven and data-driven codes (33). The theory-driven codes were derived from existing literature on Inuit and northern health. Data-driven codes were inducted directly from the preliminary planning meetings with the community and first round of interviews. Data were coded and continually refined, grouped and categorized into emergent themes relevant to understanding of the water–health relationship in the case study community. NVivo 9™ computer software was used as an organizational aid during this process. On the advice of the Community Research Liaison, a local radio call-in show was used to report and refine our preliminary analysis with Coral Harbour residents (34). This forum allowed us to gain feedback from the broader community on the plausibility of our analysis (35). Final interpretations were provided to the municipal Hamlet office and all participants in a summary report. Participants were also given the option to review their interview transcripts for accuracy, and an opportunity to review how their quotes would be used in context prior to publication (35).

## Findings

We identified three key themes associated with the role that water has in shaping health and well-being within the household and at the community level: 1) household size and water usage patterns; 2) vulnerability and coping mechanisms used to respond to water delays; and 3) water shortages as a limitation to adherence of health and sanitation advice from public health officials. We expand on these themes in the remainder of this section. A supplementary table containing participating residents’ responses related to the number of people within their home, frequency of water shortages, and basic demographics has been included to provide contextualization for their included quotes (see Table I).

**Table I:** Resident participant characteristics

Participant code	Gender	Age	Number of people per household^a^	Reported “out of water” response^b^
P-001	F	18–30	7–8	Often
P-002	F	31–40	9 +	Often
P-003	F	31–40	5–6	Often
P-004	M	18–30	9 +	Often
P-005	F	18–30	5–6	Often
P-006	M	31–40	3–4	Sometimes
P-007	F	41–49	7–8	Sometimes
P-008	M	31–40	5–6	Sometimes
P-009	F	18–30	5–6	Sometimes
P-010	F	41–49	5–6	Sometimes
P-011	F	41–49	1–2	Sometimes
P-012	F	18–30	5–6	Sometimes
P-013	F	18–30	3–4	Sometimes
P-014	F	41–49	7–8	Sometimes
P-015	M	60 +	1–2	Sometimes
P-016	F	31–40	1–2	Rarely
P-017	F	31–40	5–6	Rarely
P-018	M	18–30	1–2	Rarely
P-019	F	41–49	1–2	Rarely
P-020	M	18–30	1–2	Rarely
P-021	M	60 +	1–2	Rarely
P-022	M	31–40	5–6	Rarely
P-023	M	41–49	1–2	Rarely
P-024	F	50–59	3–4	Rarely
P-025	F	41–49	3–4	Rarely
P-026	M	41–49	1–2	Rarely
P-027	M	31–40	3–4	Rarely
P-028	M	41–49	1–2	Rarely

a Some resident participants reported that the number of people residing in their home varied day-to-day. The maximum number of people residing in their home with some regularity (defined as three or more times per month) has been recorded in this table.

b Where participants responded with “often,” it meant at least one time per week, where participants responded with “sometimes,” it meant at least one time per 2–4 weeks, and where participants responded with “rarely,” it meant one time per 3 months or less.

### Household size and water usage patterns

According to municipal water usage data we were able to estimate that, on average, each resident uses about 110 litres per capita per day. This figure is slightly above the aforementioned 100 litres per capita per day regional baseline design standard, but only one-third of the Canadian average (36). Uses for water in the household were consistent among participants and included expected consumption and hygiene activities such as drinking, food preparation, bathing, housecleaning, and laundry. However, the amount of water required or desired by each household in order to complete their day-to-day household tasks varied significantly between participants. Table I illustrates that 15 of the 28 households reported being without domestic water at least once per 2–4 weeks. Asked about the delivery schedule, which is daily or every other day in Coral Harbour given optimal conditions, one participant commented, “It's got to be daily … we'll have no water if it's not delivered on a daily basis” (P-013). Echoing this response, another participant living in a household of nine described their frequent water shortages:

If everyone showers or bathes it takes about half of the tank. I have to bathe my three youngest together. I don't like it but those are our limits … the water truck comes almost every day but I always run out of water … It wasn't always like this, not until my family grew. It's probably even more difficult if a family has more babies I bet. (P-002)

Participants did in fact report that households with young children often require more water than they receive:

I've been waiting for water since this morning [interview was conducted at approximately 4 pm]. I live in a [row house unit with a shared water tank] and all of us got babies. So maybe twice a week, three times a week, we run out of water … when it's almost gone … I always save a little bit of water for my little one in a container. That's about it. You can't use the toilet. (P-003)

Contrasting the perspective of large families with young children, a participant who lived alone with her husband, and was among the 13 of 28 residents who reported rarely experiencing shortages, explained their reduced water needs, “… I only need [water delivered] every second day” (P-019). However, the same participant empathized with larger families in overcrowded conditions, “I think it's difficult for them … sometimes they go on the radio asking to get water delivery [when municipal water operators are not immediately able to respond to a call for service]” (P-019).

### Vulnerability and coping mechanisms in response to domestic water delays

Many participants reported that municipal water delivery was prone to inconsistency and delays further straining access to water in their households. The causes of these water delays were reported as weather (particularly winter blizzards), mechanical problems with the water reservoir pumps or delivery/collection trucks, municipal holidays and water operator retention challenges.

Participants identified three coping techniques used to deal with municipal water delivery delays: 1) retrieving their own water, or ice, from local rivers and lakes, 2) relying on neighbours and extended family to share available water and 3) altering their daily activities based on water availability. For example, by independently retrieving water one participant conveyed her high tolerance to, and ability to deal with, delays, “If it's winter, [a water delay] is no problem, pick up ice. During summer, we go to one of the rivers and pick up our water ourselves” (P-017).

Relatedly, many participants conveyed their preference for independently retrieving water as per traditional practices, regardless of a delay situation, which alleviated their dependence on municipal services: “I get my own water. It's better that way” (P-015). However, participants did not report equal ability to independently collect water from rivers and lakes during delays: “it depends on if you have a vehicle or snowmobile” (P-004).

Relying on extended family and friends was the second coping mechanism. Examples of responses include: “My neighbours usually come by to get water, maybe every month … You don't even have to call, you can just walk in … We don't ‘borrow’ the water and we don't ‘return’ the water” (P-010); “In the wintertime, people pick up some ice and drop it off to me.” (P-021); and “When we are low on water, there are some friends and family just around the corner” (P-018).

Constantly re-arranging basic day-to-day activities around water delivery times was a third coping technique: “You always have to think ahead to the next day [when using water]” (P-002). Despite noting their frustration, some participants accepted that periodically not having water at your home was part of everyday life:

Six live in this house … well, [it] could be more at times when [relatives] sleep over. When there is more, we run out of water … every 2 or 3 days … It's not fun … but it's okay … when you are used to not having water. (P-004)

A key health informant acknowledged the overcrowding issues in Nunavut communities and how they interrelate with water provisions, “… A two-bedroom house that has ten people living in it – I don't think that's uncommon at all. However, just because there are more of them living in one house doesn't mean they should not have access to water.” Another key health informant captured aspects of the above-mentioned coping techniques as well as the heightened vulnerability of certain subsections of the population to water delays:

We have other means of getting water during the summer and winter too, melting ice or melting snow … we make do. But for instance, if there are people that don't have so much, like an Elder or somebody who doesn't have a husband and family around them, that for sure creates more problems because they wouldn't be able to get water supplied as much as somebody else … if they are alone in the house.

Some participants commended the Hamlet on their level of communication when dealing with water delays. In particular, their proactive messaging via community radio: “They announce that they will be a bit behind [and therefore advise residents to] conserver water” (P-023). Participants also noted, with appreciation, that the Hamlet periodically employed workers to retrieve truckloads of ice blocks from nearby water sources and deposit them in central locations throughout the community for individual household pick-up, to be melted indoors, and used for drinking and food preparation.

### In-home water shortages limit adherence to health and sanitation advice from public health officials

Domestic water delays and shortages exceeded the notion of inconvenience for some participants, and began limiting their ability to adhere to hygiene related public health practices and routines. For example, one participant discussed the challenges and resulting distress of not being able to follow advice related to her child's skin irritation diagnosis when faced with water shortages:

[The Health Centre said] … it's important to keep everything clean. You know, wash clothes and blankets and sheets often and stuff like that … it's stressful … there are limits. I can't if there is low water … There are no showers and no laundry. You have to spare water for the next day. I am never caught up. (P-002)

Based on their insight, key health informants reported specifically on *H. pylori* and MRSA as two communicable infections noticeably present in Coral Harbour, which can be passed from person-to-person in overcrowded or unhygienic housing conditions due to inadequate water usage. Specifically referring to MRSA, one key informant affirmed:

Poor hygiene … does contribute to the disease process … Part of stopping or preventing MRSA is excellent hygiene, so clothes washing, house cleaning, bathing, having your towels clean, all of that. So with that not happening, MRSA is rampant in Nunavut … it's really bad in Coral Harbour.

Another key health informant provided an additional anecdote regarding the health implications of lengthy water delays in a typical remote Nunavut community:

Hygiene, especially for people with more than four people in the house, would be a growing concern … With tuberculosis or if it's flu season and you have to use the toilet so many times a day, and you have a household [with water shortages] it could be a … disaster.

## Discussion

The current trucked water system is an improvement over complete reliance on individual-haul systems. These modifications to municipal water services in the Arctic, in conjunction with general improvements to housing conditions, have decreased the incidence of water-borne disease and transmission of communicable infectious diseases since early settlement years (37). Our research revealed, however, that many distribution system challenges still exist and that some households in the case study community are not receiving adequate quantities of municipally delivered water. As a result, their health and well-being may be negatively affected if they are not capable of supplementing their domestic water supply by other means. In particular, the consequences of shortages and interruptions are more severe for participants in overcrowded housing, families with young children and households dealing with existing communicable infections. Essentially, housing inadequacies and resulting water security are perpetuating health risks related to infectious disease transmission (10,37).

The physical environment and economic realities in Nunavut limit the options available for changes to large-scale municipal water delivery systems and infrastructure (38,39). Furthermore, our data show that community-wide changes are likely not necessary in Coral Harbour at this time as nearly 50% of our resident participants reported that they rarely ran out of domestic water. Rather, other proximal and intermediate health determinants, such as adequate housing, health care systems and individual behaviour, should be considered to improve the situation for those who did report vulnerability to water stresses (40,41). The 100 litres per capita per person water supply design standards that current municipal delivery rates are based on were informed by research that assumed homogeneity within Inuit communities and households (18–20). It is now evident that this is no longer, and perhaps never was, the case. Our research illustrates the need for engineers and community planners to consider the unique social challenges that exist in Nunavut communities when sizing household water tanks and planning water delivery services. Larger factors of safety should be used when estimating dwelling occupancy rates to account for known issues with overcrowding. Our interview data and in-situ observations show that the size of a water tank in a four-person public housing unit is standardized, but the number of people living there is not. Therefore, it is time to revisit this design standard. Water supply design standards based on actual number of occupants per household, household water tanks volumes and realistically achievable municipal water delivery schedules may be more appropriate and accurate for today's North. Furthermore, the application of such revised water supply figures, in complement with current “person per room” indicators used in housing research may strengthen approaches to addressing the health consequences of overcrowding issues in the region (41,42).

More immediate and practical public health interventions include increased municipal delivery service. Municipalities may be able to accomplish this cost-conscientiously by reviewing their delivery schedules periodically and weighting them towards larger families. Thereby larger families would receive more frequent delivery than smaller families who are satisfied with current service levels. Incremental installation of larger water storage tanks as well as water systems which safely reuse grey water (e.g. bathing wastewater for toilet flushing) within public housing units may also be effective points of action. We also suggest that consideration be given to the installation of self-serve faucets at water reservoirs, which provide easy access to treated water, and are available to residents during short delays in trucked service. Finally, systematic communication protocols between health centres and municipal water operators wherein health officials prompt additional water delivery to certain households based on their medical situation may be effective.

### Limitations

The following are limitations of our study: 1) A complete assessment of water security and public health includes aspects of access, availability, quality and stability over time (12). Within our study, however, we have purposefully created an artificial separation of access from the other components in effort to isolate and identify specific potential health risks associated with domestic water shortages and disruptions in Canada's northern Inuit communities; 2) For our purposes, we defined individually collecting surface water or ice as a coping strategy being used during municipal delivery shortages. This definition, while useful for understanding how households augment their municipal supply, does not reflect the important Inuit societal and cultural values associated with this activity; and 3) The 110 litres of water per capita per person figure was calculated by averaging the total amount of water delivered by the municipality of Coral Harbour in 2011 by the total population. We validated this figure with residents during interviews and are confident that it is a fair estimate. Since it is an average, however, we also believe that that some individuals are regularly using significantly less water per day. A research design measuring exact quantities used per individual household would allow for a more precise range of community water usage figures.

## Conclusion

This research offers a portrayal of the interconnected relationship between water and public health in a remote Arctic community. As Inuit continue to balance elements of both traditional and contemporary lifestyles, this study has uncovered a complex scenario: in a natural environment abound with fresh water sources, some families are without adequate water supply at home. Our findings show that daily water use per person in Coral Harbour is one-third the Canadian average. However, this is an estimate based on gross municipal delivery figures. Our data collected at the household level revealed that, while these relatively low domestic water quantities are adequate for some families, those living in overcrowded households are accessing water at levels more typically seen in developing countries such as India, Bangladesh and some East African nations (1). Although some people are coping by retrieving water independently or sharing between households – as is customary for Inuit – for some subsections of the population, water shortages are limiting their ability to follow public health standards and negatively impacting their overall well-being. While our findings are specific to Coral Harbour, there is some transferability to other communities given the commonalities in environmental and social conditions in northern Canada and other Arctic regions.
